# Computer Vision-Driven Movement Annotations to Advance fNIRS Pre-Processing Algorithms

**DOI:** 10.3390/s24216821

**Published:** 2024-10-24

**Authors:** Andrea Bizzego, Alessandro Carollo, Burak Senay, Seraphina Fong, Cesare Furlanello, Gianluca Esposito

**Affiliations:** 1 Department of Psychology and Cognitive Science, University of Trento, 38068 Rovereto, Italy; alessandro.carollo@unitn.it (A.C.); meiyueseraphina.fong@unitn.it (S.F.); gianluca.esposito@unitn.it (G.E.); 2 HK3 Lab, 38068 Rovereto, Italy; cesare.furlanello@hk3lab.ai

**Keywords:** neuroimaging, computer vision, deep learning, motion artifact algorithms, functional near-infrared spectroscopy, fNIRS, motion detection

## Abstract

Functional near-infrared spectroscopy (fNIRS) is beneficial for studying brain activity in naturalistic settings due to its tolerance for movement. However, residual motion artifacts still compromise fNIRS data quality and might lead to spurious results. Although some motion artifact correction algorithms have been proposed in the literature, their development and accurate evaluation have been challenged by the lack of ground truth information. This is because ground truth information is time- and labor-intensive to manually annotate. This work investigates the feasibility and reliability of a deep learning computer vision (CV) approach for automated detection and annotation of head movements from video recordings. Fifteen participants performed controlled head movements across three main rotational axes (head up/down, head left/right, bend left/right) at two speeds (fast and slow), and in different ways (half, complete, repeated movement). Sessions were video recorded and head movement information was obtained using a CV approach. A 1-dimensional UNet model (1D-UNet) that detects head movements from head orientation signals extracted via a pre-trained model (SynergyNet) was implemented. Movements were manually annotated as a ground truth for model evaluation. The model’s performance was evaluated using the Jaccard index. The model showed comparable performance between the training and test sets (*J train* = 0.954; *J test* = 0.865). Moreover, it demonstrated good and consistent performance at annotating movement across movement axes and speeds. However, performance varied by movement type, with the best results being obtained for repeated (*J test* = 0.941), followed by complete (*J test* = 0.872), and then half movements (*J test* = 0.826). This study suggests that the proposed CV approach provides accurate ground truth movement information. Future research can rely on this CV approach to evaluate and improve fNIRS motion artifact correction algorithms.

## 1. Introduction

Functional near-infrared spectroscopy (fNIRS) is a brain imaging technique that uses light to measure changes in blood oxygen levels [1]. fNIRS offers participants a greater degree of freedom of movement and is also less sensitive to movement in comparison to other techniques such as functional magnetic resonance imaging (fMRI) and electroencephalography (EEG) [2,3]. However, there are still ongoing efforts towards developing reliable methods for managing residual motion artifacts [4] that continue to potentially affect the result signal quality [5].

**Figure 1:**
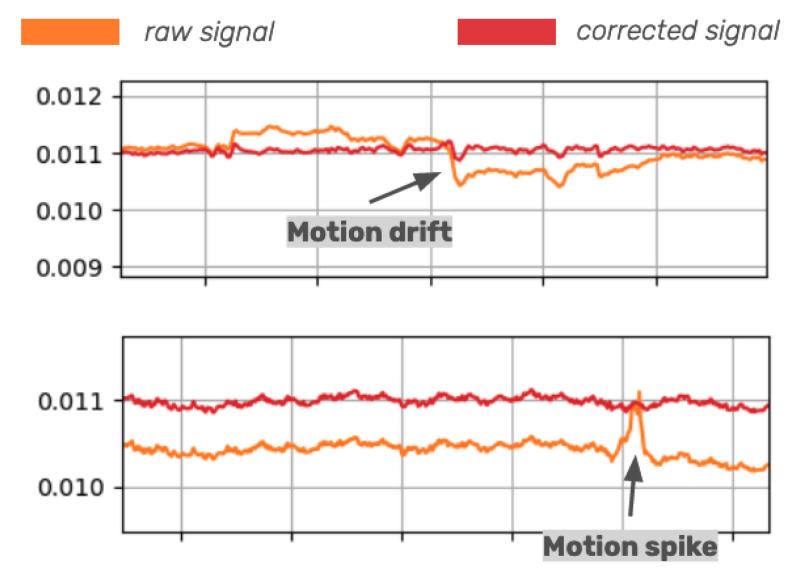
Examples of motion artifacts (motion drifts and motion spikes) in functional near-infrared spectroscopy (fNIRS) signal pre-correction (in orange) and post-correction (in red).

Two examples of motion artifacts that can occur in an fNIRS signal before and after correction are shown in Figure 1. The existence of motion artifacts in the signal can lead to spurious results if left untreated [6]. In the literature, there is no consensus on the optimal approach for addressing motion artifacts in fNIRS signals. While some researchers choose to discard the affected signals entirely, others prefer to correct the signals to retain as much data as possible [7]. Discarding artifact-affected data is not only wasteful but potentially unfeasible in situations where the experiment requires participants to move [8]. Several methods have been developed for motion artifact detection and removal (e.g., Wiener filter [9], Kalman filter [10], spline interpolation [11], wavelet filtering [12], principal component analysis [13], and temporal derivative distribution repair [6]). Spline interpolation [11] and wavelet filtering [12] are currently considered the gold-standard methods for motion artifact correction [7]. Spline interpolation is particularly effective at correcting motion drifts, while wavelet filtering is better suited for addressing motion spikes. However, these correction methods are predominantly based on theoretical assumptions regarding the expected hemodynamic brain responses or experimental outcomes. These algorithms were not developed using detailed information about the localization and type of movement, as manual annotations of movement data are highly labor-intensive. Moreover, the lack of ground truth movement information has limited the ability to evaluate the performance of these signal processing methods. For this reason, algorithm performance is typically assessed using simulated brain data rather than real ground truth information on the performed movements (e.g., [14]). Therefore, without ground truth information, it is difficult for experimenters to be certain whether these correction methods are specifically targeting motion artifacts or inadvertently removing genuine brain activity from the signal. Objective ground truth movement data are crucial for accurately evaluating the effectiveness of motion artifact correction algorithms on real fNIRS data. Furthermore, the availability of such data would enable a more robust comparison and evaluation of different pre-processing pipelines.

Three approaches can be considered to obtain ground truth information to support robust motion artifact correction. The first approach is manual annotation of movement instances within the signal or from video recordings. However, this method is both time-consuming and labor-intensive [8]. The second approach involves using a motion sensor, such as an accelerometer. For instance, Virtanen et al. [8] used accelerometer data as a measure of the participant’s head movements and developed a method for motion artifact identification and correction in fNIRS signals. Other motion artifacts correction methods were developed using accelerometer data. These methods include adaptive filtering, active noise cancellation [15], accelerometer-based motion artifact removal [8], acceleration-based movement artifact reduction algorithm [16], multi-stage cascaded adaptive filtering [17], and blind source separation accelerometer-based artifact rejection and detection [18]. However, not all fNIRS devices are equipped with accelerometers, and processing accelerometer data to detect movement requires additional algorithms. These additional algorithms can potentially complicate the analytical pipeline unless artificial intelligence (AI)-based solutions are implemented. The third alternative approach is the use of AI and computer vision (CV) to automatically detect and annotate movement instances. This approach is based on videos recorded during experimental sessions and aims to automatically detect head movements. In this way, it could potentially provide a more efficient and cost-effective solution compared to manual annotations. Manual annotation is often used in neuroimaging experiments to capture behavioral data but it is time-consuming and labor-intensive. In contrast, the AI- and CV-based approaches offer a streamlined and economical alternative that does not require additional instrumentation beyond the standard equipment typically available in laptops and smartphones.

Previous work in electrocardiogram (ECG) signal processing has successfully used UNet convolutional neural network architectures for one-dimensional semantic segmentation of ECG signals (e.g., [19,20]). In line with this, we argue that a similar AI approach can be applied to automatically detect movement instances in a signal. In the present work, we interpret the problem as a semantic segmentation task and train a 1D-UNet to identify head movements from head orientation signals extracted from videos of participants using a pre-trained SynergyNet [21]. The aim of the present work is to present a valid and reliable approach for obtaining objective movement annotations during fNIRS experimental sessions. We leverage AI and CV to automate the detection of head movements, thereby providing an efficient and cost-effective solution for obtaining ground truth movement data.

## 2. Methods

### 2.1. Study Design

Participants underwent an experiment instructing them to mimic controlled movements that were shown in a video presented on a monitor. During the experiment, participants’ brain activity was recorded using fNIRS, and the experimental session was recorded via webcam. For the scope of the current manuscript, we only utilized the webcam data and did not include the fNIRS data in the analysis. Subsequently, information on movement was extracted from the videos *(i)* using a CV approach and *(ii)* with manual annotations.

Data collection was approved by the University of Trento’s ethical research committee (2023-054). Experimental sessions followed the guidelines provided by the Declaration of Helsinki. Informed consent was obtained from all participants.

### 2.2. Participants

Fifteen participants were recruited for the current work using convenience and snowball sampling from social media sites and through the University of Trento’s participant management and recruitment SONA system. Participants could take part in the experiment if they were at least 18 years old and if they had no history of known and/or diagnosed health or neurological conditions.

The sample size aligns with previous technical work in fNIRS research (e.g., Lanka et al. [22], Santosa et al. [23]). We did not perform a formal sample size calculation based on power analysis because we were not testing specific hypotheses.

### 2.3. Experimental Procedure

**Figure 2:**
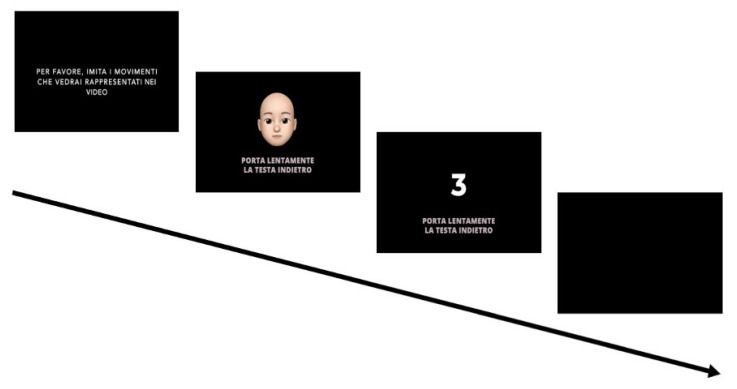
Experimental paradigm. Participants were instructed to mimic head movements displayed in video stimuli. The video began by prompting participants to imitate the moments presented. An avatar was then shown demonstrating the movements with a text caption below it describing the demonstrated action, followed by a 3 s countdown before 7 s of requested head movement.

The experimental paradigm is depicted in Figure 2. Participants were asked to mimic a series of head movements in a video displayed on a monitor. The presentation of the instructional stimuli was controlled with *PsychoPy*, and all instructions were provided in Italian. The experimental video began with an instructional segment, prompting participants with the following instructions: “Please, imitate the movements represented in the video”. Subsequently, an avatar demonstrated the movements with a text description of the action at the bottom of the screen (e.g., “Move your head backward slowly”). This was carried out via a 3 s countdown. Participants were then given 7 s to perform the instructed head movement.

In the experimental video, the avatar demonstrated two categories of movements. These categories included *(i)* head movements and *(ii)* facial expressions. Head movements were executed along three axes (i.e., head up/head down, head left/head right, bend head left/bend head right) at both fast and slow speeds. Each movement involved both partial and full rotations (i.e., half, complete, and repeated movements). For example, participants were instructed to move their head and gaze upwards in one instance (half movement), tilt their head left and right in another instance (complete movement), and turn their heads from left to right multiple times (repeated movement). The sequence was repeated three times. Consequently, the entire experiment encompassed a total of 60 head movements.

### 2.4. fNIRS Data Acquisition

**Figure 3:**
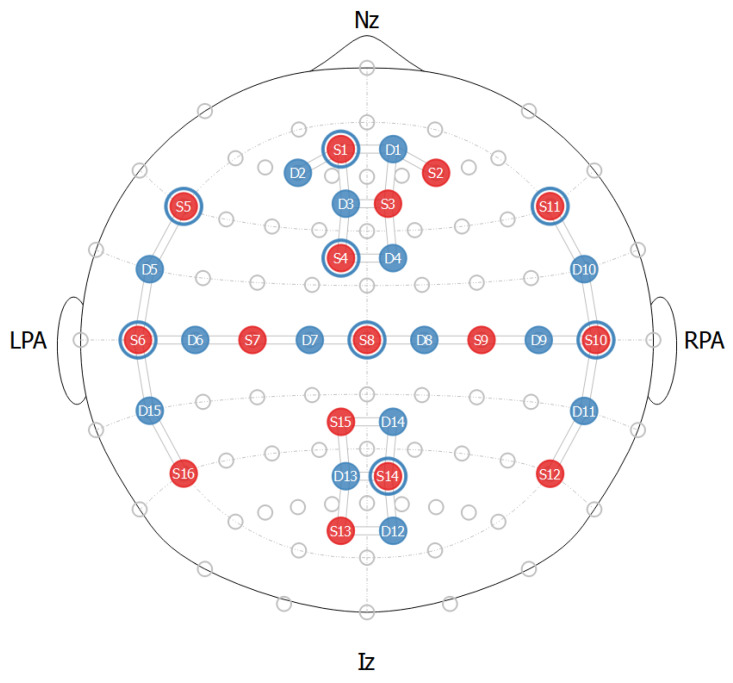
Illustration of the functional near-infrared spectroscopy (fNIRS) cap setup employed in this study. Light sources are marked in red, while detectors are marked in blue. The blue circles surrounding the light sources indicate short-distance channels.

Participants’ brain activity was monitored using fNIRS, with each cap consisting of 16 LED sources that emitted NIR light at wavelengths of 760 nm and 850 nm, paired with 16 light detectors. One of these detectors was specifically assigned to collect signals from eight short-distance channels. The optode placement followed the standard international 10–20 electroencephalography layout, providing comprehensive coverage of the participants’ brain activity. This configuration resulted in a total of 32 channels (see Figure 3). Optode stabilizers were used to ensure both a consistent distance between the sources and detectors (i.e., never exceeding 3 cm) and a good signal-to-noise ratio [24]. The fNIRS data were collected using a NIRSport2 device (NIRx Medical Technologies LLC) at a sampling rate of 10.17 Hz.

For the purpose of the current work (i.e., to present a computer vision approach for accurate motion detection), the fNIRS data were not used in the analysis.

### 2.5. Deep Learning Approach

Two deep learning models were implemented in the present work: SynergyNet [21] and a one-dimensional UNet model (1D-UNet).

#### 2.5.1. SynergyNet

SynergyNet is a pre-trained model that can extract head position (three axes) and head orientation (three axes) information [21]. The network establishes a synergistic process between 3D Morphable Models (3DMM) and 3D facial landmarks to predict 3D facial geometry [21]. It consists of two key modules: (1) multi-attribute feature aggregation to refine facial landmarks with MobileNet-V2 [25] as the backbone, and (2) mapping 3D landmarks back to 3DMM parameters, enabling the regression of embedded facial geometry from sparse landmarks. Further details about SynergyNet’s model implementation can be found in the original paper [21] and GitHub (https://github.com/choyingw/SynergyNet, website accessed on 23 October 2024).

In the present paper, we utilize the pre-trained SynergyNet to process the frames of each participant’s webcam video to only obtain head orientation information. The detected head orientation data from all video frames were concatenated to obtain the three components of the head orientation signal.

#### 2.5.2. UNet

Following the success of previous work using UNet convolutional neural network architectures for the one-dimensional semantic segmentation of ECG signals (e.g., [20]), we trained a 1D-UNet for semantic segmentation of the head orientation signals to identify instances of motion. UNet models are commonly used for image segmentation tasks. They can effectively capture both local and global features due to their “U-shaped” architecture [26]. The architecture consists of two main components: a contracting path for feature extraction and an expansive path for precise localization of the target to segment. Skip connections are used to retain information from earlier layers and therefore enhance the model’s performance. Overall, UNets are known for achieving good accuracy even with limited training data. This makes them suitable for various applications with scarce labeled data (e.g., biomedical imaging and other domains [20,26]).

The present work’s 1D-UNet was trained on movement onset annotations from video recordings of 10 randomly selected participants. Data from the remaining five participants were used for testing. As a standard practice in machine learning (e.g., [27]), the training–test partition was used to assess the model’s generalizability by evaluating its performance on unseen data.

Using the PyTorch framework [28], the architecture of the current work’s 1D-UNet model closely followed that of Moskalenko et al. [20]. This consistency ensured that we implemented the effective feature extraction and segmentation capabilities demonstrated in previous studies.However, the number of channels in each convolutional and deconvolutional level was modified in order to tailor the model to the specific characteristics of the input data and task requirements. Specifically, the number of channels of the 1D-Unet used in this study was as follows: 16, 32, 64, 128, and 256 (instead of 4, 8, 16, 32, and 64, used in [20]). The 1D convolutional layers had a kernel size of 9, stride size of 1, and padding size of 4. The last convolutional layer had a kernel size of 1. The model was trained with the Adadelta optimization algorithm [29], dice loss function [30], batch size of 64, learning rate of 0.1, and for 1000 epochs.

The 1D-UNet architecture implemented in this paper consists of the following:

1. **Input Layer:** The network accepts a 1D input signal.
2. **Contracting Path (Encoder):** The encoder is composed of five blocks. Each block contains the following: (a) two 1D convolutional layers (kernel size = 9, stride = 1, padding = 4), followed by batch normalization and ReLu activation, and (b) a max-pooling layer (pool size = 2) for downsampling.
3. **Bottleneck:** The bottleneck consists of two 1D convolutional layers that are each followed by batch normalization and ReLU activation.
4. **Expanding Path (Decoder):** The decoder mirrors the encoder and also consists of five blocks. Each block contains the following: (a) upsampling of feature maps using transposed convolution or *nn.Upsample* with scale factor = 2; (b) concatenation with the corresponding feature maps from the encoder path; and (c) two 1D convolutional layers with batch normalization and ReLU activation.
5. **Output Layer:** The output is produced with a 1D convolutional layers (kernel size = 1). This reduces the number of channels to 1 for the purpose of binary classification. A sigmoid activation function is applied to the output to obtain the binary classification result.
6. Xavier and Kaiming methods for weight initialization.

### 2.6. Model Evaluation

Instances of movement were manually annotated from head orientation signals by two independent raters. The annotations were then validated against the videos and used as ground truth information for model performance evaluation.

The Jaccard index was used to evaluate the performance of the trained 1D-UNet model as it is commonly used in the evaluation of semantic segmentation models [31]. It is defined in Formula 1, where A∩B represents the intersection of sets A and B, and A∪B represents the union of sets A and B.

(1) $$J\left(A,B\right)=\frac{\left|A\cap B\right|}{\left|A\cup B\right|}$$

The Jaccard index (*J*) measures the similarity (overlap) between the ground truth movements (*A*) and the model’s predicted (*B*) movements. A score of 1 indicates maximum similarity between the movement annotations. This score is therefore the best possible performance attainable by the model as its movement predictions are in line with the ground truth movement information [31]. To obtain a more reliable indication of the identification performance, bootstrap performance values are reported. Specifically, the 2.5–97.5% confidence intervals are computed using the scikits-bootstrap Python package.

## 3. Results

**Figure 4:**
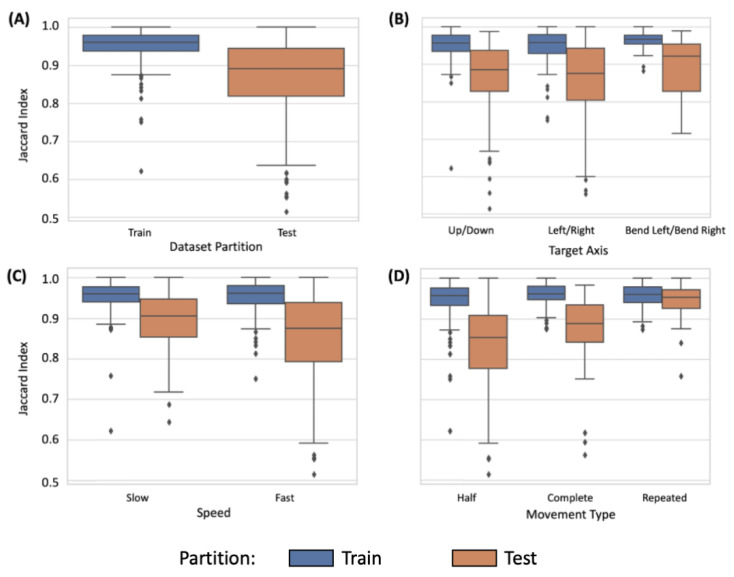
One-dimensional U-Net (1D-UNet) model performance evaluated using the Jaccard index on (**A**) dataset partition type (training versus test set), (**B**) target movement axis, (**C**) movement speed, and (**D**) movement type.

Figure 4 depicts the performance of the 1D-UNet model across various factors. Firstly, the 1D-Unet had good and comparable performance across the training (*J* = 0.954 [0.949, 0.958]) and test set partitions (*J* = 0.865 [0.847, 0.878]; Figure 4A). Moreover, similar performance for the training and test sets was observed across target movement axes (Figure 4B; Up/Down: Train *J* = 0.951 [0.943, 0.957], Test *J* = 0.862 [0.836, 0.883], Left/Right: Train *J* = 0.952 [0.945, 0.958], Test *J* = 0.854 [0.826, 0.878], BendLeft/BendRight: Train *J* = 0.965 [0.960, 0.970], Test *J* = 0.893 [0.868, 0.916]) and movement speeds (Figure 4C; Slow: Train *J* = 0.953 [0.944, 0.959], Test *J* = 0.892 [0.874, 0.908], Fast: Train *J* = 0.955 [0.949, 0.959], Test *J* = 0.847 [0.826, 0.868]). However, the results suggest an influence of movement type (half, complete, repeated, Figure 4D) on the model’s performance. In particular, better performance was observed for repeated movements (Train *J* = 0.956 [0.950, 0.962], Test *J* = 0.941 [0.925, 0.953]), followed by complete (Train *J* = 0.960 [0.953, 0.965], Test *J* = 0.872 [0.839, 0.894]) and finally half movements (Train *J* = 0.950 [0.942, 0.956], Test *J* = 0.826 [0.805, 0.848]). Hence, the CV model provided accurate information on ground truth movements, which was comparable to the manual annotations.

## 4. Discussion

fNIRS has a high tolerance for movement when compared to other neuroimaging techniques. However, the presence of motion artifacts in fNIRS signals is still an issue. This is because the signal processing methods typically used to perform motion artifact correction do so and are evaluated without ground truth movement information. To address this gap of ground truth information, three approaches can be considered: manual annotation of movement, using motion sensors like accelerometers [8], or employing AI-based CV techniques to automatically detect movements from video recordings. Given that manual annotation is time-consuming and not all fNIRS devices have built-in accelerometers, the current study aims to utilize a CV approach to annotate ground truth motion data from head orientation signals, offering a more efficient and accessible solution. The significance of this work lies in identifying a feasible and accurate method for extracting objective ground truth movement data. This information can subsequently serve as a basis for evaluating and refining fNIRS motion artifact correction strategies.

In our experiment, fifteen participants were instructed to perform controlled movements (e.g., quickly/slowly turn head left/right/up/down or tilt head left/right) while being recorded with a webcam. The results indicate that segmentation models, such as a 1D-UNet model, have the potential to effectively annotate movements from head orientation signals extracted from videos using the pre-trained SynergyNet [21]. As compared to manual annotations, the 1D-UNet model demonstrated good performance across various aspects of movement detection, including dataset partitions (train/test set), target movement axes (up/down, left/right, bend left/bend right), and movement speeds (slow/fast). However, detecting the type of movement (i.e., half, complete, repeated) showed more variability. These results suggest that the model’s performance may potentially further benefit from additional movement annotation data, additional raters, and refined manual annotation techniques with inter-rater agreements to ensure quality and consistency among ground truth annotations. Moreover, this study is based on a sample of 15 participants, which aligns with similar research in the field (e.g., [22,23]). While we acknowledge that the sample size is still limited, we urge for the adoption of similar approaches across different labs, which not only would provide a larger sample size but also help ensure and test the model’s accuracy and generalizability across different contexts and populations.

Future research could also consider fine-tuning or re-training the model on naturalistic movements (i.e., where participants move their heads across multiple rotational axes). It is also worth noting that the head orientation signals obtained through SynergyNet are comparable to the data collected from an accelerometer. Thus, future research could apply the 1D-UNet to different types of movement data. This would enhance the generalizability of the model beyond controlled lab environments and sources for obtaining head orientation signals. Another option would be to use the movement information to predict the occurrence of motion artifacts in an fNIRS signal. This may enable the pre-processing of fNIRS data to be more robust as it could further minimize the loss of actual brain activity while effectively identifying and removing motion artifacts.

Overall, there is a need for standardized practices to ensure the replicability of fNIRS studies across laboratories [5,32]. Despite some algorithms demonstrating good performance in motion artifact correction, the lack of ground truth movement information continues to hinder the evaluation and refinement of these approaches. An automated ground truth movement detection method based on actual and video-recorded head movements could be highly valuable for researchers working on standardizing and evaluating fNIRS pre-processing pipelines. This study shows that CV can extract ground truth information from experimental session videos without external sensors or extra signals.

## Data Availability

Data are available upon request from the corresponding author.

## Abbreviations

The following abbreviations are used in this manuscript:

1D-UNet	one-dimensional U-Net
AI	artificial intelligence
CV	computer vision
ECG	electrocardiogram
EEG	electroencephalography
fNIRS	functional near-infrared spectroscopy
fMRI	functional magnet resonance imaging
